# Is repetitive systemic corticosteroid therapy effective for idiopathic sudden sensorineural hearing loss? a retrospective study

**DOI:** 10.3389/fneur.2023.1167128

**Published:** 2023-04-28

**Authors:** Kengo Yamamoto, Takaomi Kurioka, Motofumi Ohki, Hajime Sano, Taku Yamashita

**Affiliations:** ^1^Department of Otorhinolaryngology, Kitasato University Medical Center, Kitamoto, Japan; ^2^Department of Otorhinolaryngology-Head and Neck Surgery, Kitasato University School of Medicine, Sagamihara, Kanagawa, Japan; ^3^Department of Otorhinolaryngology, National Defense Medical College, Tokorozawa, Japan; ^4^School of Allied Health Science, Kitasato University, Sagamihara, Kanagawa, Japan

**Keywords:** idiopathic sudden sensorineural hearing loss, hearing prognosis, prognostic factor, corticosteroid therapy, sudden deafness

## Abstract

**Introduction:**

Some idiopathic sudden sensorineural hearing loss (ISSHL) cases experience repetitive systemic corticosteroid treatment, but studies focusing on repetitive systemic corticosteroid administration have not been reported. Thus, we investigated the clinical characteristics and usefulness of repetitive systemic corticosteroid treatment in ISSHL cases.

**Methods:**

We reviewed the medical records of 103 patients who received corticosteroids only in our hospital (single-treatment group), and 46 patients who presented at our hospital after receiving corticosteroids in a nearby clinic and were subsequently treated with corticosteroids again in our hospital (repetitive-treatment group). Clinical backgrounds, hearing thresholds, and hearing prognosis were assessed.

**Results:**

The final hearing outcomes were not different between the two groups. Further, in the repetitive-treatment group, statistical differences were found between the good and poor prognosis groups in the number of days to start corticosteroid administration (*p* = 0.03), the dose of corticosteroid (*p* = 0.02), and the duration of corticosteroid administration (*p* = 0.02) at the previous facility. Multivariate analysis revealed a significant difference in the dose of corticosteroids administered by the previous clinic (*p* = 0.004).

**Conclusion:**

The repetitive systemic corticosteroid administration might play a supplementary role in hearing improvement, and initial sufficient corticosteroid administration would lead to good hearing outcomes in an early phase of ISSHL.

## Introduction

Idiopathic sudden sensorineural hearing loss (ISSHL) is usually defined as an acute unilateral sensorineural hearing loss (1). The etiology of ISSHL remains unknown, and various hypotheses have been proposed, including microcirculation disorders, viral infection, and autoimmunity (2, 3). Systemic corticosteroid administration is the mainstream of standard treatment (4), but with only 30–60% of patient responses (5). Additionally, systemic corticosteroid therapy sometimes carries the risk of serious side effects (3) and systemic management would be required. In some cases, the intensity of systemic corticosteroid treatment is needed to be weakened or even suspended depending on general health conditions of the ISSHL patient. However, systemic corticosteroid treatment has no standardized protocol among institutions regarding doses, administration route, and duration. Therefore, some patients with ISSHL may have been treated with inadequate protocol of corticosteroid administration, which resulted in poor recovery. Thus, we hypothesize that these inadequately treated cases showing poor hearing recovery could be improved by readministrating adequate dose of corticosteroid repetitively, but to the best of our knowledge, no study has reported repetitive corticosteroid treatment for initial-treatment failure patients yet.

The primary goal of medical treatment for ISSHL is to restore hearing thresholds, and better prognostic factors of ISSHL have been reported as young age, short days between onset and the start of treatment, absence of vertigo (4, 6, 7), and better hearing thresholds at onset (8). Additionally, a recent report revealed that early response to systemic corticosteroid treatment correlates with final prognosis (7). Therefore, we hypothesized that we could improve the final hearing outcome for patients with ISSHL who do not achieve early hearing recovery under primary systemic corticosteroids by intensifying the conventional treatments. However, the efficacy of repeated systemic corticosteroid administration for patients with ISSHL as an additional consolidated treatment is unclear. This study investigated the hearing outcomes of affected ear and prognostic factors in patients with ISSHL who were treated with repetitive systemic corticosteroids.

## Materials and methods

### Study design

This retrospective study was approved by the Institutional Review Board of Kitasato University Medical center (2021004). The need for informed consent was omitted owing to the retrospective nature of the study.

### Patients

This study included 149 patients hospitalized and treated for ISSHL in our hospital from 2016 to 2020 who were divided into the single-treatment group (103 patients who received corticosteroids only in our hospital) and the repetitive-treatment group (46 patients who presented to our hospital after receiving corticosteroids in a nearby clinic and were subsequently treated with corticosteroids again in our hospital). We defined ISSHL as a sudden sensorineural hearing loss of 30 dB or greater in at least three consecutive frequencies and pathogen was unidentified. Patients with acute low-tone sensorineural hearing loss, fluctuating hearing loss, any history of otologic surgery, and acoustic neuroma were excluded. We primarily judged the need for hospitalization based on symptoms, such as dizziness and severity, or a history of diabetes mellitus.

### Hearing test

Pure-tone audiometry was performed in a soundproof room. The hearing thresholds were measured through air conduction at frequencies of 0.125, 0.25, 0.5, 1, 2, 4, and 8 kHz and bone conduction at frequencies of 0.25–4 kHz for both ears. The arithmetic average air conduction thresholds were obtained from the thresholds at 0.25, 0.5, 1, 2, and 4 kHz. The severity of hearing loss grade was determined by the Japanese Ministry of Health and Welfare guidelines, using the initial audiogram data (Table 1). Hearing recovery was calculated as the difference between the average hearing thresholds at different time points. The evaluation of hearing recovery was based on the hearing outcome criteria proposed by the Acute Severe Hearing Loss Study Group of the Ministry of Health, Labor, and Welfare of Japan (Table 2). The severity of hearing loss and the evaluation of hearing recovery were obtained from the average thresholds of 0.25–4 kHz.

**Table 1:** The severity of hearing loss grade by the guidelines of the Japanese ministry of health and welfare.

Severity	
Grade1	Averaged PTA thresholds of <40 dB
Grade2	Averaged PTA thresholds of 40–60 dB
Grade3	Averaged PTA thresholds of 60–90 dB
Grade4	Averaged PTA thresholds of ≥90 dB

Averaged PTA thresholds were obtained from the average air conduction thresholds of 0.25–4 kHz. PTA, pure-tone audiometry.

**Table 2:** Final treatment outcomes according to the guideline of the Acute Severe Hearing Loss Study Group of the Ministry of Health, Labor, and Welfare of Japan.

Description	
Complete recovery	All five frequencies at 0.25, 0.5, 1, 2, and 4 kHz of final audiograms are ≤20 dB, or improvement to the same degree of hearing in the unaffected ear
Marked recovery	Averaged PTA improvement of ≥30 dB
Slight recovery	Averaged PTA improvement of 10–30 dB
No recovery	Averaged PTA improvement of <10 dB

Averaged PTA improvement was calculated as the difference between average hearing thresholds of 0.25–4 kHz at different time points, including pre-and post-treatment. PTA, pure-tone audiometry.

Audiometry was completed in our department in the single-treatment group, and the tests were performed three times: before the systemic corticosteroid administration, during corticosteroid titration, and more than 3 months following treatment, or the ISSHL is judged as fully recovered. Additionally, patients in the repetitive-treatment group underwent audiometry three times, but the first tests were measured by previous clinics. The other two tests were measured in our department before the repetitive-treatment and more than 3 months after treatment or the ISSHL is judged as fully recovered.

Patients in the repetitive-treatment group were accordingly classified into the following two groups: the good (i.e., complete and marked recovery) and the poor prognosis groups (i.e., slight and no recovery). Additionally, we investigated the prognostic factors in repetitive corticosteroid treatment.

### Treatment

We administered a 10 day course of systemic corticosteroids as a standard treatment in our institution (8 mg of betamethasone via intramuscular injection for the first day followed by 4 mg of betamethasone via oral administration for the first 3 days, tapered to 2 mg for the second 3 days and 1 mg for the last 3 days). To enhance the efficacy of ISSHL treatment, we also prescribed prostaglandin E1 (60 μg daily), vitamin B12 (1.5 mg daily) and adenosine triphosphate (300 mg daily). The corticosteroid administration started by a previous physician was terminated in the repetitive-treatment group, and then the same protocol as in the single-treatment group was started at our department. Details of corticosteroid treatment attempted by a previous physician were shown in Supplementary Table.

### Assessment

Individual clinical features and examination results, including age at onset, gender, the severity of hearing loss, presence of vertigo, time from the onset to the start of initial treatment, and time from the onset to the start of treatment in our hospital, were investigated. Additionally, we investigated the protocol of corticosteroid therapy performed by a nearby clinic in the repetitive-treatment group.

### Statistical analyses

Statistical analysis was conducted using GraphPad Prism 8 (GraphPad Software Inc., La Jolla, CA, United States) or JMP 14.2 (SAS Institute Japan Inc., Tokyo, Japan). We used the chi-squared test to evaluate the clinical characteristics and possible prognostic factors. The *t-*test or nonparametric Mann–Whitney *U* test was applied to investigate continuous variable prognostic factors. The difference in hearing thresholds was analyzed using a two-way analysis of variance followed by Šidák’s multiple comparison tests. After univariate analysis, we included various parameters that were statistically significant in the univariate analysis into a binary logistic regression model for multivariate analysis. A *value of p* of <0.05 was considered statistically significant.

## Result

### Backgrounds

First, no cases interrupted the repetitive corticosteroid treatment due to the serious side effects in the repetitive-treatment group. Additionally, patients in the repetitive-treatment group were significantly younger (63.5 years vs. 54.5 years, *p* = 0.002), but with no statistically significant differences in gender, the severity of hearing loss, or the presence of vertigo. The start of treatment in our department was significantly delayed (5.0 days vs. 8.6 days, *p* < 0.0001) because of the pre-treatment period at a nearby clinic although the time to start treatment was shorter in the repetitive-treatment group (5.0 days vs. 3.6 days, *p* = 0.01). No statistical difference was detected in the duration from onset to post-treatment hearing examination between the groups (Table 3).

**Table 3:** Patient backgrounds of the two groups.

	Single-treatment group (*N* = 103)	Repetitive-treatment group (*N* = 46)	*p*
Age (years)	63.5	54.5	***0.002***
Gender (male/ female)	59/44	23/23	*0.41*
Severity (Grade 1/2/3/4)	12/27/38/26	7/11/20/8	*0.66*
Presence of vertigo (+/−)	28/75	15/31	*0.50*
Days to start primary treatment	5.0	3.6	***0.01***
Days to start treatment in our department	5.0	8.6	***<0.0001***
Duration from onset to final hearing evaluation (weeks)	19.03	18.61	0.658

Bold indicates significant differences (< 0.05).

**Figure 1:**
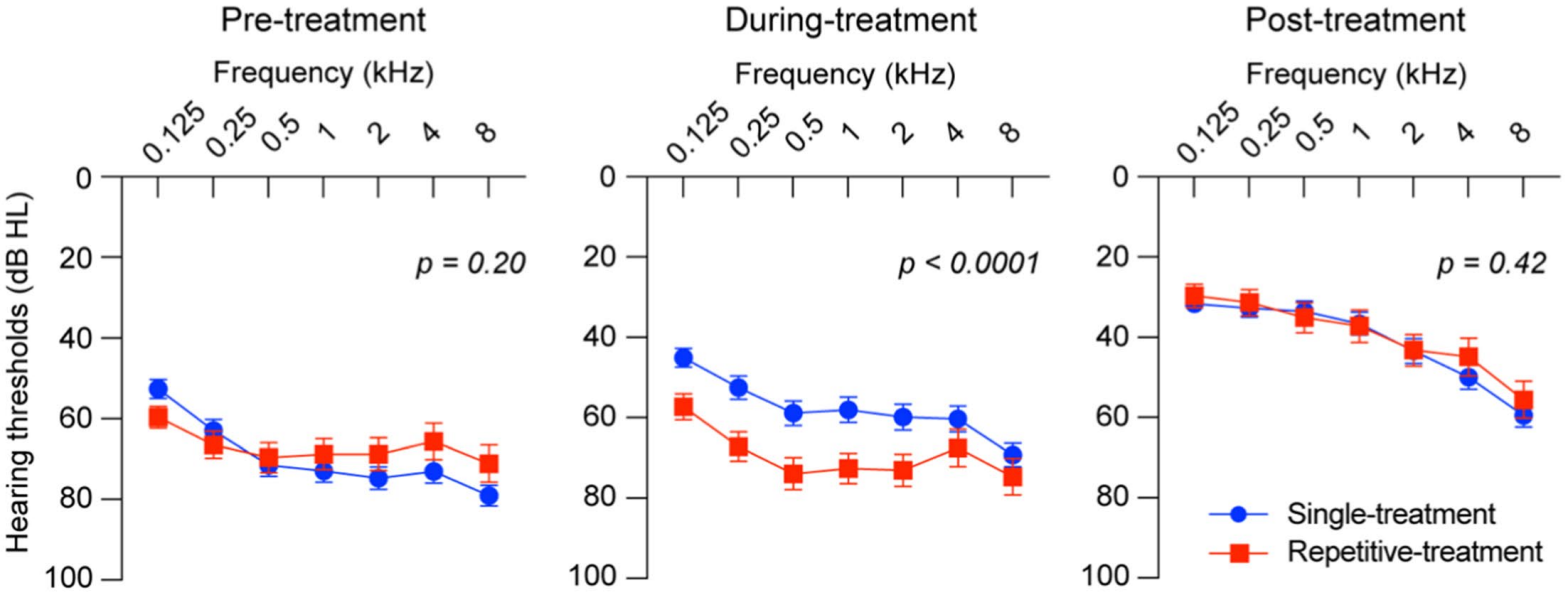
Hearing thresholds of the single-and repetitive-treatment groups. Hearing thresholds were significantly different between groups at during-treatment but not pre- and post-treatment. Bars represent standard error of the mean.

The hearing thresholds of the two groups at pre-treatment, during treatment, and post-treatment are shown at every measured frequency (Figure 1). No statistical difference was found between the two groups in the hearing thresholds at the measurement of pre-treatment and post-treatment, indicating poor early response to initial corticosteroid treatment and slower hearing recovery in the repetitive-treatment groups than those of the single-treatment group although the repetitive-treatment group revealed significantly worse hearing thresholds during treatment.

### Prognostic factors in the repetitive-treatment group

Prognostic factors in the repetitive-treatment group were further investigated by dividing 46 patients into two groups: good (24 patients) and poor prognosis groups (22 patients). Statistical differences were found in the number of days to start corticosteroid administration at a previous facility (2.5 days vs. 4.8 days, *p* = 0.03), the dose of corticosteroid in methylprednisolone (mPSL) equivalent (0.44 mg vs. 0.33 mg, *p* = 0.02) and the duration of corticosteroid administration (2.8 days vs. 4.0 days, *p* = 0.02), indicating patients in the poor prognosis group were treated later and received a smaller dose of corticosteroids at a previous clinic. Additionally, the start of corticosteroid administration in our department was significantly delayed in the poor prognosis group due to the previous facility treatment periods (6.1 days vs. 11.1 days, *p* = 0.003). In particular, the dose of corticosteroids administered by a previous clinic was significantly different on multivariate analysis (*p* = 0.004) (Table 4).

**Table 4:** Prognostic factors in the repetitive-treatment group.

	Good prognosis group	Poor prognosis group	*p*	
	(*N* = 24)	(*N* = 22)	Univariate	Multivariate
Age	52.5	56.8	*0.39*	
Gender(male/female)	10/14	13/9	*0.24*	
Severity (grade 1/2/3/4)	2/4/12/6	5/7/8/2	*0.19*	
Days to start treatment in the previous facility	2.5	4.8	***0.03***	*0.37*
Days to start treatment in our department	6.1	11.1	***0.003***	*0.25*
Dose of corticosteroid in the previous facility (equivalent to mPSL; mg/kg)	0.44	0.33	***0.02***	***0.004***
Duration of corticosteroid treatment in the previous clinic (days)	2.8	4.0	***0.02***	*0.06*

Bold indicates significant differences (< 0.05).

**Figure 2:**
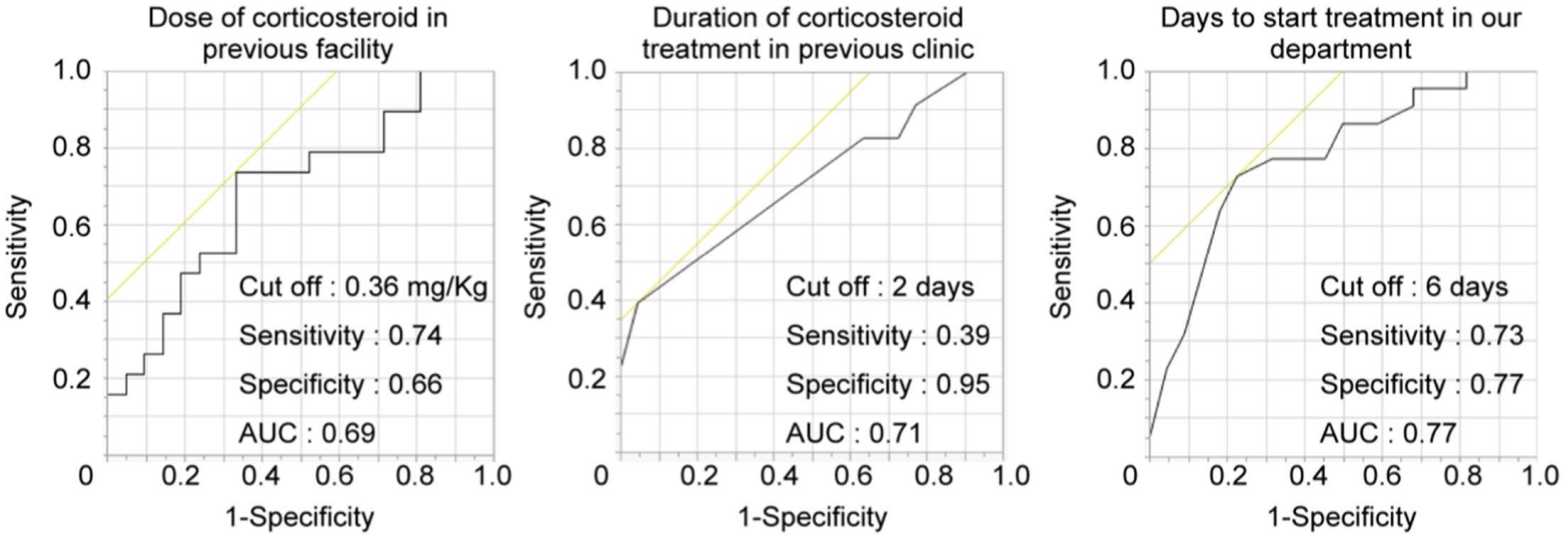
Cut-off value of prognostic factors in the repetitive-treatment group. ROC curve to predict the hearing prognosis according to corticosteroid dose, duration, and days to start treatment. The AUCs were 0.69, 0.71, and 0.77, respectively. AUC, area under the curve.

These results revealed that the timing and dose of corticosteroid administration at the previous clinic affected the prognosis of ISSHL. Therefore, we further calculated the cut-off value from the ROC curve to elucidate the effect of primary corticosteroid administration at the previous clinic. Cut-off values of the corticosteroid dose were 0.36 mg per kg of body weight (sensitivity: 0.74, specificity: 0.66, area under the curve [AUC]: 0.69), the duration of administration at the previous doctor was 2 days (sensitivity: 0.39, specificity: 0.95, AUC: 0.71), and the start date of re-initiation at our department was 6 days from the onset (sensitivity: 0.73, specificity: 0.77, AUC: 0.77) (Figure 2).

## Discussion

Various therapeutic strategies for ISSHL are proposed in addition to systemic corticosteroid administration, which is considered one of the standard treatments worldwide. Intratympanic corticosteroid injection (9, 10) could deliver high concentration of corticosteroid to inner ear (11) without serious systemic side effects, and are recognized as one of the effective salvage treatments (12). Moreover, hyperbaric oxygen therapy (HBOT), which improves microcirculation by increasing oxygen concentration in inner ear (13), is also a therapeutic option for salvage in severe ISSHL (14). However, similar to systemic corticosteroid administration that has the risk of general side effects (15), intratympanic corticosteroid injections and HBOT also rarely, but occasionally have some risk of dizziness (16), persistent tympanic membrane perforation (17), inner ear injury occurred in 17.3% patients (18) and resultant hearing improvement was limited (19). Therefore, physicians may hesitate to prescribe large doses of corticosteroids systemically to all ISSHL cases without adequate medical care equipment, such as clinics, even to try intratympanic injection for salvage. Conversely, we often diagnosed patients with ISSHL who were initially treated with systemic corticosteroids at a nearby clinic and consulted our hospital for seeking additional treatment and examination because of poor hearing improvement. This consulting situation in Japan was considered for some reasons; some ISSHL cases recover slowly, and the policy of the national insurance system promotes segregation between hospitals and clinics. At present, there has been no established and standardized salvage treatment for ISSHL and proposed salvage therapies have some disadvantages, as mentioned above. To the best of our knowledge, no study has been reported focusing on the hearing outcome of repetitive systemic corticosteroid administration in patients with ISSHL. This is the first study to investigate the hearing outcomes of affected ear and its therapeutic characteristics in patients with ISSHL treated with repeated systemic corticosteroid therapy.

In our study, although no significant difference was observed in the hearing thresholds before and after treatment between the single-and repetitive-treatment groups, the hearing thresholds during the treatment was statistically different. The two groups tracked different recovery processes of hearing recovery, considering the difference in the number of days between the two groups until the start of treatment. It may be because of the difference in ISSHL pathogenesis between the two groups. However, patients in the repetitive-treatment group first visited other clinics and consulted our hospital for further detailed inspection and additional treatment. We hypothesized that patients with ISSHL with relatively slow hearing recovery accumulated in the repetitive-treatment group due to a selection bias because approximately 10% of patients with ISSHL recover their hearing even after >3 months (20) from ISSHL onset. Concluding the effects of repetitive corticosteroids treatment is difficult based on our study alone, but repetitive systemic administration of steroids did not hinder hearing recovery, thereby suggesting systemic repetitive corticosteroids treatment might be recommended as a choice of salvage therapy for ISSHL under certain conditions, such as in patients who are hesitant to receive intratympanic steroid injection, or in facilities where HBOT is not equipped. Then, we investigated therapeutic characteristics in the repetitive-treatment group and revealed significant differences in the number of days from the onset to the start of treatment and the initial dose of corticosteroid administered at nearby clinic between poor and good prognosis groups. Large doses of corticosteroids are considered necessary to elicit the efficacy of corticosteroids for inner ear pathology because the more systemic corticosteroids are prescribed, the more corticosteroids reach the inner ear (21). Initial treatment, especially sufficient corticosteroid administration in the early stage of onset, would make a significant contribution to hearing recovery, considering starting treatment within 7 days of onset is associated with a good prognosis (2) and the effectiveness of treatment is less likely to be obtained after 2 weeks of onset as consistent with previous reports (22). The comparable final hearing outcome in the repetitive-treatment group and the single-treatment group may be the result of the initial corticosteroid administration with a time lag. Therefore, we considered the repetitive systemic corticosteroid administration to play only a supplementary role in hearing improvement.

Our results indicated that an initial dose of corticosteroids should be sufficient and should be administered as early as possible after the onset of hearing loss. Moreover, repetitive systemic corticosteroid administration might be promising strategies as additional salvage treatment for ISSHL. The results of this study may serve as a guide to identifying patients with ISSHL who can be managed as an outpatient, while inpatient treatment may be restricted due to the COVID-19 pandemic. Additionally, the accumulation of ISSHL cases with different recovery time course is expected to lead to the subdivision of ISSHL as a syndrome and identify new pathogenesis or prognostic factors of ISSHL.

Finally, our study has several limitations. First, this was a retrospective study conducted in a single hospital, and the sample size was relatively small because we only chose hospitalized cases. Second, the repetitive-treatment group was younger and the time until the start of corticosteroid administration was shorter; thus, these factors may have modified the treatment outcome. Third, the dose and type of corticosteroid administrated by a previous physician are varied.

## Conclusion

This retrospective study was conducted to determine whether repetitive systemic corticosteroid administration contributes to better hearing outcomes in patients with ISSHL, and investigate prognostic factors in the repetitive-treatment group. We concluded that sufficient and early corticosteroid administration would lead to good hearing outcomes in ISSHL although the effectiveness of repetitive systemic corticosteroid treatment remained unclear.

## Data availability statement

The original contributions presented in the study are included in the article/Supplementary material, further inquiries can be directed to the corresponding author.

## Ethics statement

The studies involving human participants were reviewed and approved by the Institutional Review Board of Kitasato University Medical center. Written informed consent for participation was not required for this study in accordance with the national legislation and the institutional requirements.

## Author contributions

KY and TK designed this study, analyzed the results, and prepared the manuscript. TK performed the analysis. KY and MO included and treat the patients. HS and TY improved the manuscript. All authors contributed to the article and approved the submitted version.

## Funding

The study was supported by the grant from “Japan society for the promoting of science” and “Daiwa Securities Health Foundation”.

## Conflict of interest

The authors declare that the research was conducted in the absence of any commercial or financial relationships that could be construed as a potential conflict of interest.

## Publisher’s note

All claims expressed in this article are solely those of the authors and do not necessarily represent those of their affiliated organizations, or those of the publisher, the editors and the reviewers. Any product that may be evaluated in this article, or claim that may be made by its manufacturer, is not guaranteed or endorsed by the publisher.

## References

[1] Staecker H Jokovic G Karpishchenko S Kienle-Gogolok A Krzyzaniak A Lin CD. Efficacy and safety of am-111 in the treatment of acute unilateral sudden deafness-a double-blind, randomized, placebo-controlled phase 3 study. Otol Neurotol. (2019) 40:584–94. doi: 10.1097/MAO.0000000000002229, PMID: 31083077 PMC6553962

[2] Watanabe H Sano H Maki A Ino T Nakagawa T Okamoto M. Investigation of stress levels before the onset of idiopathic sudden sensorineural hearing loss. J Int Adv Otol. (2019) 15:51–5. doi: 10.5152/iao.2019.6197, PMID: 31058595 PMC6483419

[3] Merchant SN Adams JC Nadol JB Jr. Pathology and pathophysiology of idiopathic sudden sensorineural hearing loss. Otol Neurotol. (2005) 26:151–60. doi: 10.1097/00129492-200503000-00004, PMID: 15793397

[4] Kitoh R Nishio SY Ogawa K Kanzaki S Hato N Sone M. Nationwide epidemiological survey of idiopathic sudden sensorineural hearing loss in Japan. Acta Otolaryngol. (2017) 137:S8–S16. doi: 10.1080/00016489.2017.1297537 28394652

[5] Qiang Q Wu X Yang T Yang C Sun H. A comparison between systemic and Intratympanic steroid therapies as initial therapy for idiopathic sudden sensorineural hearing loss: A Meta-analysis. Acta Otolaryngol. (2017) 137:598–605. doi: 10.1080/00016489.2016.1260157, PMID: 27921520

[6] Kim JY Han JJ Sunwoo WS Koo JW Oh SH Park MH. Sudden sensorineural hearing loss in children and adolescents: clinical characteristics and age-related prognosis. Auris Nasus Larynx. (2018) 45:447–55. doi: 10.1016/j.anl.2017.08.010, PMID: 28888426

[7] Shimanuki MN Shinden S Oishi N Suzuki N Iwabu K Kitama T. Early hearing improvement predicts the prognosis of idiopathic sudden sensorineural hearing loss. Eur Arch Otorhinolaryngol. (2021) 278:4251–8. doi: 10.1007/s00405-020-06532-4 33389010

[8] Kurioka T Sano H Furuki S Yamashita T. Long-term administration of vitamin B12 and adenosine triphosphate for idiopathic sudden sensorineural hearing loss: A retrospective study. PeerJ. (2020) 8:e10406. doi: 10.7717/peerj.10406, PMID: 33362960 PMC7749652

[9] Ahmadzai N Kilty S Cheng W Esmaeilisaraji L Wolfe D Bonaparte JP. A systematic review and network Meta-analysis of existing pharmacologic therapies in patients with idiopathic sudden sensorineural hearing loss. PLoS One. (2019) 14:e0221713. doi: 10.1371/journal.pone.0221713, PMID: 31498809 PMC6733451

[10] Spear SA Schwartz SR. Intratympanic steroids for sudden sensorineural hearing loss: A systematic review. Otolaryngol Head Neck Surg. (2011) 145:534–43. doi: 10.1177/0194599811419466 21873598

[11] Parnes LS Sun AH Freeman DJ. Corticosteroid pharmacokinetics in the inner ear fluids: an animal study followed by clinical application. Laryngoscope. (1999) 109:1–17. PMID: 10399889 10.1097/00005537-199907001-00001

[12] Kakehata S Sasaki A Futai K Kitani R Shinkawa H. Daily short-term Intratympanic dexamethasone treatment alone as an initial or salvage treatment for idiopathic sudden sensorineural hearing loss. Audiol Neurootol. (2011) 16:191–7. doi: 10.1159/000320269 20962524

[13] Lamm K Lamm C Arnold W. Effect of isobaric oxygen versus hyperbaric oxygen on the Normal and noise-damaged hypoxic and ischemic Guinea pig inner ear. Adv Otorhinolaryngol. (1998) 54:59–85. doi: 10.1159/000059054, PMID: 9547878

[14] Eryigit B Ziylan F Yaz F Thomeer H. The effectiveness of hyperbaric oxygen in patients with idiopathic sudden sensorineural hearing loss: A systematic review. Eur Arch Otorhinolaryngol. (2018) 275:2893–904. doi: 10.1007/s00405-018-5162-6, PMID: 30324404 PMC6244669

[15] Rudmik L Soler ZM. Medical therapies for adult chronic sinusitis: A systematic review. JAMA. (2015) 314:926–39. doi: 10.1001/jama.2015.7544 26325561

[16] Liu YC Chi FH Yang TH Liu TC. Assessment of complications due to Intratympanic injections. World J Otorhinolaryngol Head Neck Surg. (2016) 2:13–6. doi: 10.1016/j.wjorl.2015.11.001, PMID: 29204543 PMC5698530

[17] Topf MC Hsu DW Adams DR Zhan T Pelosi S Willcox TO. Rate of tympanic membrane perforation after Intratympanic steroid injection. Am J Otolaryngol. (2017) 38:21–5. doi: 10.1016/j.amjoto.2016.09.004, PMID: 27751619

[18] Hadanny A Meir O Bechor Y Fishlev G Bergan J Efrati S. The Safety of Hyperbaric Oxygen Treatment--Retrospective Analysis in 2,334 Patients. Undersea Hyperb Med. (2016) 43:113–22. 27265988

[19] Ng JH Ho RC Cheong CS Ng A Yuen HW Ngo RY. Intratympanic steroids as a salvage treatment for sudden sensorineural hearing loss? A Meta-analysis. Eur Arch Otorhinolaryngol. (2015) 272:2777–82. doi: 10.1007/s00405-014-3288-8, PMID: 25217083

[20] Yeo SW Lee DH Jun BC Park SY Park YS. Hearing outcome of sudden sensorineural hearing loss: long-term follow-up. Otolaryngol Head Neck Surg. (2007) 136:221–4. doi: 10.1016/j.otohns.2006.10.021 17275543

[21] Kanzaki S Watanabe K Fujioka M Shibata S Nakamura M Okano HJ. Novel in vivo imaging analysis of an inner ear drug delivery system: drug availability in inner ear following different dose of systemic drug injections. Hear Res. (2015) 330, no. Pt A:142–6. doi: 10.1016/j.heares.2015.09.018 26435094

[22] Amarillo E Navarro A Hernandez-Garcia E Plaza G. Intratympanic steroids for combined treatment of idiopathic sudden hearing loss: when is it too late? Acta Otolaryngol. (2019) 139:632–5. doi: 10.1080/00016489.2019.1614222 31124732

