# Supplementary material for: Is repetitive systemic corticosteroid therapy effective for idiopathic sudden sensorineural hearing loss? a retrospective study
Source: Front Neurol. 2023 Apr 28;14:1167128. doi: 10.3389/fneur.2023.1167128 (PMC10175769; doi:10.3389/fneur.2023.1167128)
Supplement: Supplementary file 1 [file table_1.docx]

**Supplementary Table**

Initial doses of corticosteroid treatments administered by a previous physician.

| Initial doses of corticosteroids at previous clinic | Case(s) |
| --- | --- |
| Dexamethasone 10mg | 1 |
| Dexamethasone 8mg | 1 |
| Dexamethasone 4mg | 29 |
| Methylprednisolone 60mg | 2 |
| Methylprednisolone 40mg | 2 |
| Methylprednisolone 30mg | 5 |
| Methylprednisolone 20mg | 1 |
| Methylprednisolone 10mg | 1 |
| Methylprednisolone 5mg | 1 |
| Unknown | 3 |
